# Validity of the Polar Vantage M watch when measuring heart rate at different exercise intensities

**DOI:** 10.7717/peerj.10893

**Published:** 2021-02-09

**Authors:** Tricia Shumate, Magdalen Link, James Furness, Kevin Kemp-Smith, Vini Simas, Mike Climstein

**Affiliations:** 1 Department of Physiotherapy, Faculty of Health Science and Medicine, Bond University, Gold Coast, QLD, Australia; 2 Water Based Research Unit, Bond University, Gold Coast, QLD, Australia; 3 Clinical Exercise Physiology, School of Health and Human Sciences, Southern Cross University, Bilinga, QLD, Australia; 4 Physical Activity, Lifestyle, Ageing and Wellbeing, Faculty Research Group, Faculty of Health Sciences, University of Sydney, Lidcombe, NSW, Australia

**Keywords:** Wrist worn fitness tracker, Exercise, Bruce protocol, Polar vantage watch, Heart rate, Intensity, Validity, Exercise science

## Abstract

**Background:**

The use of wrist worn wearable fitness trackers has been growing rapidly over the last decade. The growing popularity can be partly attributed to the improvements in technology, making activity trackers more affordable, comfortable and convenient for use in different fitness and environmental applications. Fitness trackers typically monitor activity level, track steps, distance, heart rate (HR), sleep, peripheral capillary oxygen saturation and more, as the technology continuously is advancing. In terms of measuring HR, photoplethysmography (PPG) is a relatively new technology utilised in wearables. PPG estimates HR through an optical technique that monitors changes in blood volume beneath the skin. With these new products becoming available it is important that the validity of these devices be evaluated. Therefore, the aim of this study was to assess the validity of the Polar Vantage M (PVM) watch to measure HR compared to medical grade ECG on a healthy population during a range of treadmill exercise intensities.

**Methods:**

A total of 30 healthy participants (*n* = 17 males, *n* = 13 females) were recruited for this study. The validity of the PVM watch to measure HR was compared against the gold standard 5-lead ECG. The study was conducted on 2 separate testing days with 24–48 h between sessions. Participants completed the Bruce Treadmill Protocol, and HR was measured every 30 s. Validation of the PVM watch in comparison to the ECG was measured with an Intraclass Correlation Coefficient (ICC) and associated 95% confidence intervals (CI) and levels of agreement were identified with Bland–Altman plots with 90% limits of agreement. Linear regression analysis was performed to calculate the value of *r*^2^ computing the variation of HR obtained by the PVM watch and ECG.

**Results:**

In total, 30 participants completed the protocol, with data from 28 participants utilised for statistical analysis (16 males, 14 females, 26.10 ± 3.39 years, height 52.36 m ± 7.40 cm, mass 73.59 ± 11.90 kg). A strong and significant correlation was found between the PVM watch and ECG, demonstrating good criterion validity (*p* < 0.05, *r*^2^ = 0.87). Good validity was seen for day 1 and day 2 for stage 0 (ICC = 0.83; 95% CI [0.63–0.92], ICC = 0.74; 95% CI [0.37–0.88]), stage 1 (ICC = 0.78; 95% CI [0.52–0.90], ICC = 0.88; 95% CI [0.74–0.95]), and stage 2 (ICC = 0.88; 95% CI [0.73–0.94], ICC = 0.80; 95% CI [0.40–0.92]). Poor validity was demonstrated on day 1 and day 2 for stages 3–5 (ICC < 0.50).

**Conclusion:**

This study revealed that the PVM watch had a strong correlation with the ECG throughout the entire Bruce Protocol, however the level of agreement (LoA) becomes widely dispersed as exercise intensities increased. Due to the large LoA between the ECG and PVM watch, it is not advisable to use this device in clinical populations in which accurate HR measures are essential for patient safety; however, the watch maybe used in settings where less accurate HR is not critical to an individual’s safety while exercising.

## Introduction

The use of wrist worn wearable fitness trackers has been growing rapidly over the last decade (Phaneuf, 2019). People across all demographics are using fitness trackers to measure and analyse physical activity and body functions of the wearer (Pothitos, 2016). Fitness trackers typically monitor activity level, track steps, distance, heart rate (HR), sleep, peripheral capillary oxygen saturation (SpO_2_), and more, as the technology continuously is advancing (Pothitos, 2016). Based on a recent Australian market analysis by Statista Global Consumer Survey, the revenue from wearables was estimated to be approximately $173 million in 2019 and the number of wearable users is expected to increase to 2.1 million by 2023 (Statista, 2020). Consumers across all age groups are expressing rising interest in this field (Statista, 2020). In 2019, 31.9% of the users were between 25 and 34 years old, which identifies the target market (Statista, 2020). The market is expanding not only in Australia, but globally with wearable technology topping the worldwide fitness trends since 2016 (Thompson, 2015; Thompson, 2019). The growing popularity can be partly attributed to the improvements in technology, making activity trackers more affordable, comfortable and convenient for use in different fitness and environmental applications (Phaneuf, 2019).

In terms of measuring HR, photoplethysmography (PPG) is a relatively new technology utilised in wearables. PPG estimates HR through an optical technique that monitors changes in blood volume beneath the skin (Shin, Shin & Lee, 2011). The ability of these watches to measure important cardiovascular parameters such as SpO_2_, HR, and heart rate variability (HRV) indicates that thorough research must be conducted on this topic to determine their reliability and validity (Alharbi et al., 2019). It is imperative to inform clinicians who might prescribe these devices and populations with medical conditions on the validity and reliability of these devices. If data from these monitors are to be used to monitor or guide patient activity and therapy, the monitors’ accuracy must be validated (Etiwy et al., 2019).

Currently, peer reviewed research with the aim to assess the validity of these devices to measure HR spans across many brands including multiple models of the Fitbit, Apple Watch, Garmin, Mio FUSE, TomTom and other brands (Etiwy et al., 2019; Abt, Bray & Benson, 2018; Bai et al., 2018; Boudreaux et al., 2018; Cadmus-Bertram et al., 2017; Claes et al., 2017; Collins et al., 2019; Dooley, Golaszewski & Bartholomew, 2017; Gillinov et al., 2017; Gorny et al., 2017; Haghayegh et al., 2019; Hernando et al., 2018; Jo et al., 2016; Khushhal et al., 2017; Lee & Gorelick, 2011; Leth et al., 2017; Nelson & Allen, 2019; Pope et al., 2019; Powierza et al., 2017; Reddy et al., 2018; Shcherbina et al., 2017; Stahl et al., 2016; Stove et al., 2019; Tedesco et al., 2019; Thiebaud et al., 2018; Thomson et al., 2019; Wang & Fu, 2016). Most of those studies involve healthy adults performing a treadmill protocol using an electrocardiogram (ECG) as a reference standard (Cadmus-Bertram et al., 2017; Claes et al., 2017; Gillinov et al., 2017; Lee & Gorelick, 2011; Leth et al., 2017; Nelson & Allen, 2019; Powierza et al., 2017; Shcherbina et al., 2017; Thiebaud et al., 2018; Thomson et al., 2019). Many studies reported good-excellent validity as defined by Intraclass Correlation Coefficients (ICC) > 0.60 or a Pearson Correlation Coefficient value of *r* > 0.50 (Etiwy et al., 2019; Abt, Bray & Benson, 2018; Bai et al., 2018; Cadmus-Bertram et al., 2017; Claes et al., 2017; Collins et al., 2019; Dooley, Golaszewski & Bartholomew, 2017; Gillinov et al., 2017; Gorny et al., 2017; Hernando et al., 2018; Khushhal et al., 2017; Powierza et al., 2017; Shcherbina et al., 2017; Stahl et al., 2016). For relative measures, the following criteria were used ICC: Poor = ICC < 0.40, Fair = ICC 0.40–0.59, Good = ICC 0.60–0.74, Excellent ≥ 0.75 (Fleiss, Levin & Paik, 2003); for *r*: negligible *r* = 0–0.29, low *r* = 0.30–0.49, moderate *r* = 0.50–0.69, high *r* = 0.70–0.89, very high *r* = 0.90–1 (Mukaka, 2012). Measurements of HR tended to be more valid at low treadmill intensities (Boudreaux et al., 2018; Gorny et al., 2017; Jo et al., 2016; Reddy et al., 2018) however one study demonstrated good validity at high intensities (12.1 km/h) (Stove et al., 2019). Limitations of the current research include varying devices tested, exercise protocols utilised, intensities tested, reference standards used, and inconsistent statistical analyses performed. Due to vast variation in devices tested, protocols used and statistical interpretation, results on validity cannot be generalised to all fitness trackers.

One of the more recent fitness trackers on the market released in September 2018, is the Polar Vantage M (PVM) watch. It is advertised as a high performance watch that ‘measures HR from the wrist with the Precision Prime™ sensor fusion technology which combines optical HR measurement with other sensor technologies in order to rule out involuntary movement that might disturb the HR signal and produce unreliable readings’ (Polar USA, 2020). Despite this claim, no peer reviewed studies have been conducted on the validity of the PVM watch to date. Therefore, the aim of this study was to assess the validity of the PVM watch to measure HR compared to medical grade ECG on a healthy population during a range of exercise intensities.

## Materials and Methods

### Study design

The current study was an observational design assessing the validity of measuring HR by the Polar Vantage M watch during incremental treadmill exercise. To assess the validity of measuring HR using the Polar Vantage M watch, participants were asked to attend the human performance laboratory on two occasions separated by 1–2 days.

### Participants

A convenience sample of 30 healthy and physically active participants (*n* = 17 males, *n* = 13 females) were recruited from a university student population through advertised electronic and printed posters on the University campus, as well as through social media. Research has provided evidence that a sample size of at least 15–20 is considered adequate for reliability studies which collect continuous data and therefore the current sample size is justified (Lexell & Downham, 2005). Ethical approval was granted through Bond University Human Research Ethics committee (ML01928). Informed consent was obtained from all participants via a signed consent form prior to participation.

Participants were excluded from participation if they had any of the following criteria: under the age of 18, any pre-existing respiratory, cardiovascular, or metabolic conditions, taking medications that affected their HR, pregnant, or any musculoskeletal injuries, current or within the last 6 weeks such as low back pain, ankle sprain, osteoarthritis, etc. Patients were also excluded if they refused to give informed consent to perform the protocol or were unable to answer ‘no’ to all six questions in the Exercise and Sport Science Australia ESSA (ESSA) Adult Pre-Exercise Screening Form (Essa.org.au, 2019).

### Instrumentation

The PVM watch (Polar Electro, Kempele, Finland) was used to compare HR measurements to the gold standard ECG while exercising at different intensities on a treadmill. A real time 7-lead (I, II, III, AvR, AvL, AvF, V5) ECG was utilised with the Mortora XScribe Cardiac Stress Test System (Serial Number: NO8200) which is a regulated medical device with intended use in clinical settings (Welch Allyn, 2018). The following five electrodes were placed respectively following standard anatomical landmarks: right arm (RA), left arm (LA), left leg (LL), right leg (RL) and V5 (chest). The PVM watch is a multi-sport watch that uses optical HR tracking. The PVM watch uses an optical measurement called photoplethysmography (PPG), which identifies volumetric changes in the microvascular structure of tissue. PPG then uses a signal that the optical HR solution measures to interpret and calculate HR (Polar Australia, 2020). During testing, the mode ‘running’ was utilised to help validate the watches’ function and accuracy. The Bruce Protocol was conducted on a Valiant Lode BV Treadmill Type: 932900 (Serial Number:20060061). The Bruce protocol (Queensland Health, 2019) was chosen because it is a standard graded exercise test, commonly used for clinical and laboratory-based assessments of cardiorespiratory fitness in athletes and patients. Additionally, the Bruce Protocol has been used in similar validation studies (Bruce, Kusumi & Hosmer, 1973) and allows for easy comparison of results against past, and future investigations.

### Experimental procedures

Participants were contacted prior to testing via mobile phone to ensure exclusion criteria did not apply. The participants were required to visit the laboratory on two separate occasions. Each participant completed both tests within 24–48 h and at approximately the same time of day. These requirements aimed to reduce any possible variance caused by change in fitness status and circadian variation.

**Figure 1:**
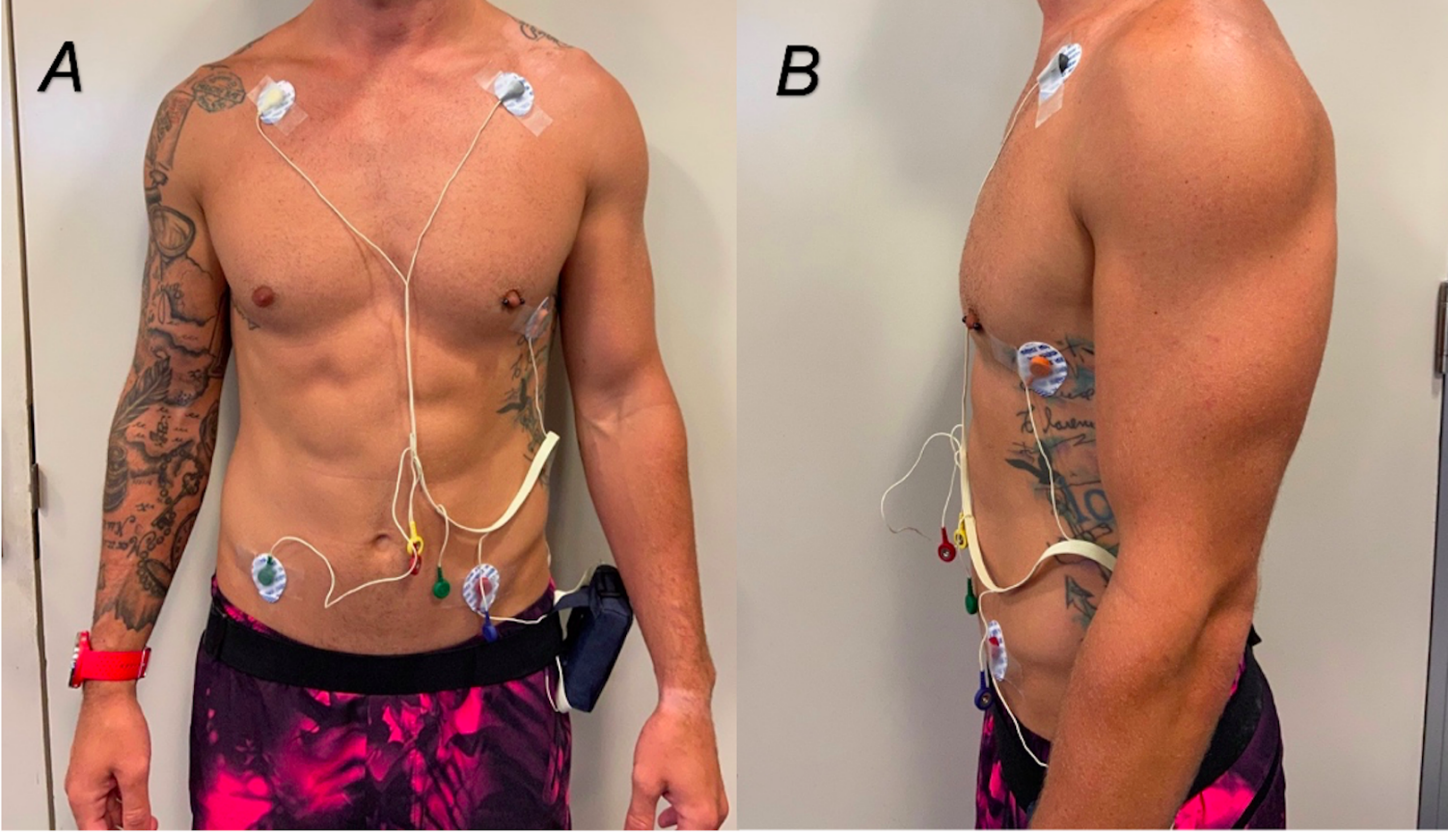
(A and B) Placement of ECG electrodes on participants.

On the first day of testing, each participant was introduced to the laboratory and required to complete the ESSA Adult Pre-Exercise Screening Tool and consent form. Personal information was collected including name, age, as well as anthropometric data including height and mass were recorded. Participants were then prepped for the ECG placement (Fig. 1). To ensure consistency, the PVM watch was placed on all participants right hand. The device’s display was oriented away from the participant to allow the researcher to easily read the HR. The tightness of the PVM watch was standardised for each patient with the number of watchband notches recorded and repeated for the second day of testing. Resting HR was recorded from the ECG and PVM watch as the participant was seated for a quiescent period for 3 min.

Heart rate readings were taken from both ECG and PVM watch every 30 s throughout the entire treadmill protocol. To obtain resting HR, the protocol started with the participants sitting stationary in a chair for 3 min, followed by 2 min of standing on the treadmill. Next, the standard Bruce Protocol was conducted increasing both treadmill speed (Stage 1, 2.7 km/h; Stage 2, 4.0 km/h; Stage 3, 5.4 km/h; Stage 4, 6.7 km/h; Stage 5, 8.0 km/h) and incline (starting at 10% grade and increasing 2% every Stage) every 3 min until volitional exhaustion (Queensland Health, 2019). Participants were not allowed to hold onto the handrail throughout the duration of the protocol, however, instructions of ‘hand’ were communicated 5 s prior to each 30 s reading to allow accurate data collection by the researcher. When participants indicated they had reached their volitional exhaustion, displayed any criteria for terminating test based upon Queensland Health indications for terminating exercise testing (Queensland Health, 2019), or ECG read the participant had attained their age-predicted maximal HR, participants were instructed to place both hands onto the handrail whilst the treadmill was put into the active cooldown stage.

### Statistical analyses

Statistical analyses were performed using SPSS (Version 24.0; IBM Corp, Armonk, NY, USA). To determine if anthropometric data was normally distributed visual inspection of the histograms, normal Q–Q plots and a Shapiro–Wilks test was conducted to determine the appropriate descriptive statistics (mean or, median). A scatter plot of combined data for Day 1 and Day 2 was used to depict the strength of the relationship between ECG measured HR and PVM watch measured HR for different intensities with the associated *r*^2^ value. To assess criterion validity between Polar and ECG HR data for Day 1 and Day 2, Intraclass correlation Coefficients (ICC) were used. Historically Koo & Mae (2017). According to Koo & Li (2016) the model type and definition should be reported when using an ICC. Applying the guidelines the model used within this statistical analysis was two-way mixed effects, the type used was the average measures and the definition used was the absolute agreement. The interpretation of the ICCs for validity were defined as the following: an ICC value of less than 0.50 indicated poor validity; ICC values in the range of 0.50–0.65 indicated moderate to good validity, and an ICC value of greater than 0.65 identified good validity (Fleiss, Levin & Paik, 2003). We presented the level of agreement between the PVM watch and ECG through Bland–Altman plots with the associated 90% limits of agreement calculated. The the equation used to determine limits of agreement (LoA) was:

LoA = mean difference ± 1.65 × (standard deviation of difference) (Bland & Altman, 1986).

## Results

### Demographics

In total, 30 participants completed the protocol, with data from 28 participants utilised for statistical analysis (16 males, 14 females, 26.10 ± 3.39 years, height 52.36 m ± 7.40 cm, mass 73.59 ± 11.90 kg). One participant was removed from data analysis due to a sinus tachycardia which was discovered following testing which significantly influenced their HR results. Another participants’ data was an extreme outlier with over 40 bpm error margin between EGC and Polar Watch HR was removed. Average HR_max_, (HRM) as calculated by 220-age, was 193.93 ± 3.39 bpm and average resting HR, as measured while sitting upright in a chair for 3 min was 71.49 ± 10.29 bpm. Percent HR_max_ was calculated for stages 0–5 for day 1 using the equation: (average HR for stage/total average HR_max_) × 100. Percentage of HRmax for stage 0–5 were found to be 43%, 53%, 61%, 79%, 93%, and 95% respectively. Interpretations for percent intensities were made following The American College of Sports Medicine (ACSM) guidelines for exercise prescription where low intensity is defined HRM < 55%, moderate intensity as 55–75% and high intensity as HRM > 75% (Deborah Riebe, Liguori & Magal, 2018). Therefore, the Bruce Protocol stages 0–1 were classified as low intensity, stage 2 as moderate intensity and stages 3–5 as high intensity.

### Criterion validity

**Figure 2:**
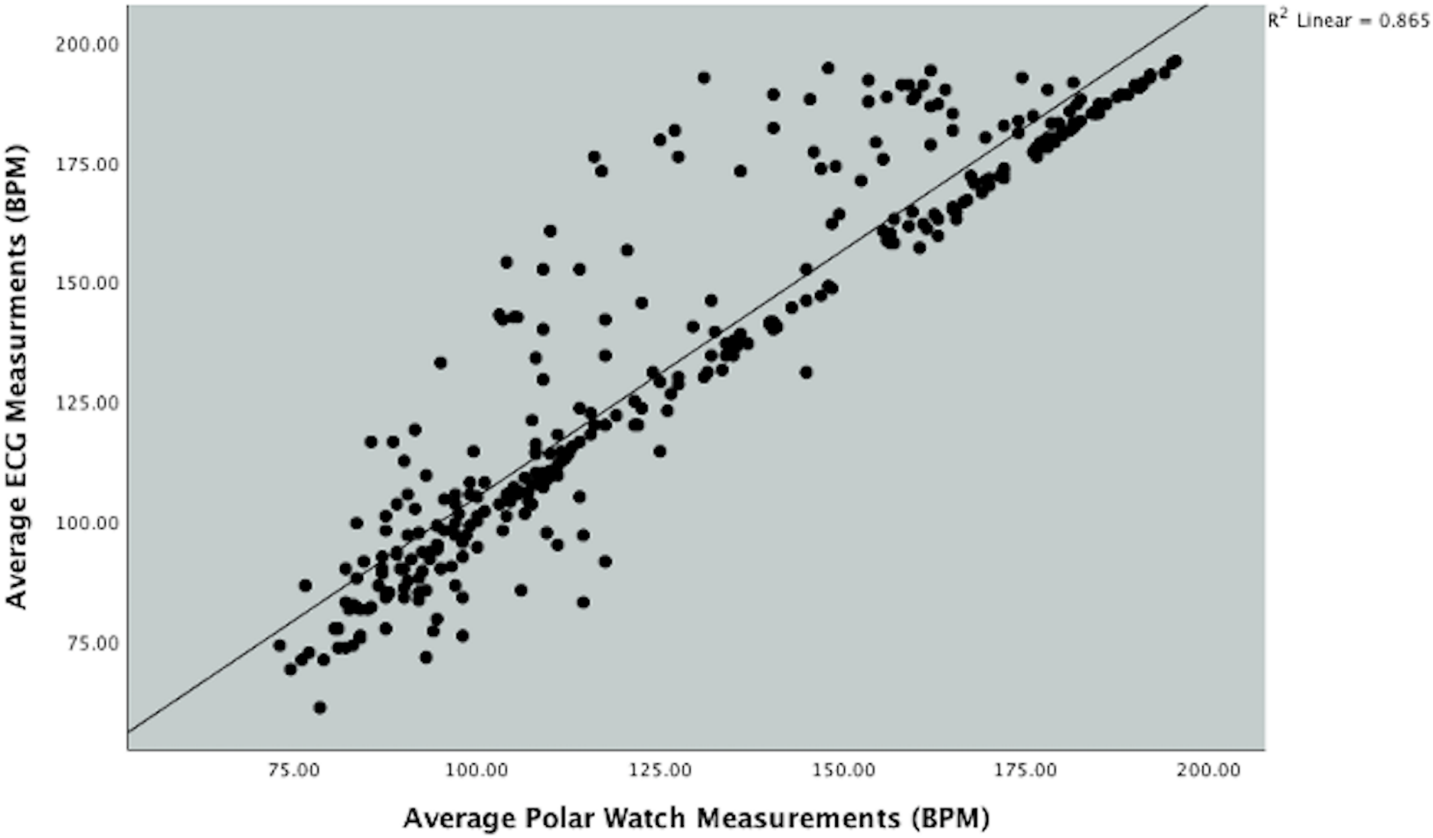
Scatterplot showing the relationship between Polar Vantage watch and ECG HR measurement during the Bruce protocol.

Figure 2 depicts the positive linear association between the ECG and PVM watch. Linear regression analysis was performed which calculated the value of *r*^2^ = 0.87, which demonstrated that approximately 87% of variation of HR obtained by the PVM watch can be explained by variation in HR obtained from the ECG.

**Figure 3:**
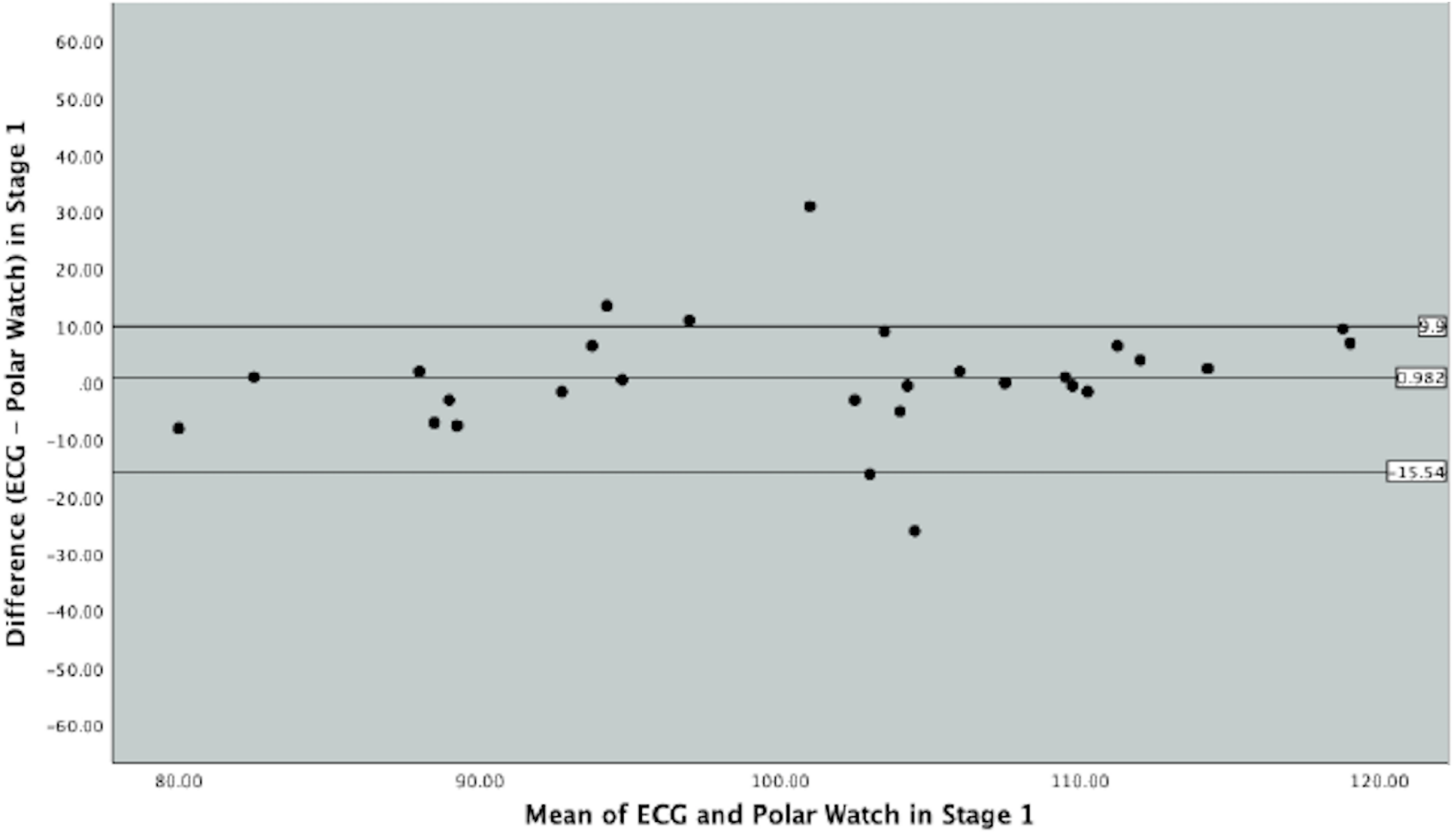
Bland–Altman Plot indicating mean difference and 90% limits of agreement between measurements from the Polar Vantage watch and ECG for HR measurement in Stage 1 of the Bruce Protocol.

**Figure 4:**
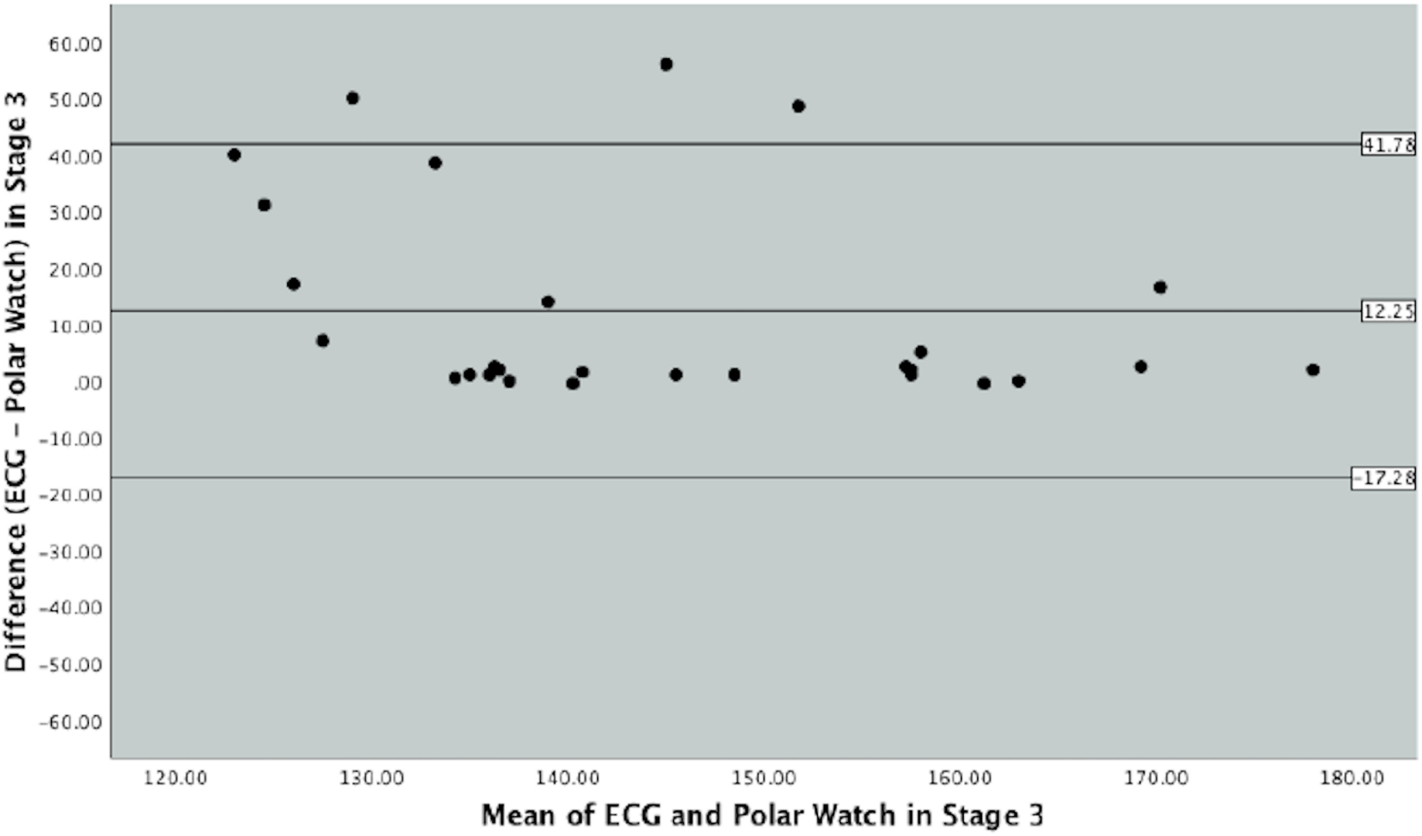
Bland–Altman Plot indicating mean difference and 90% limits of agreement between measurements from the Polar Vantage watch and ECG for HR measurement in Stage 3 of the Bruce Protocol.

**Figure 5:**
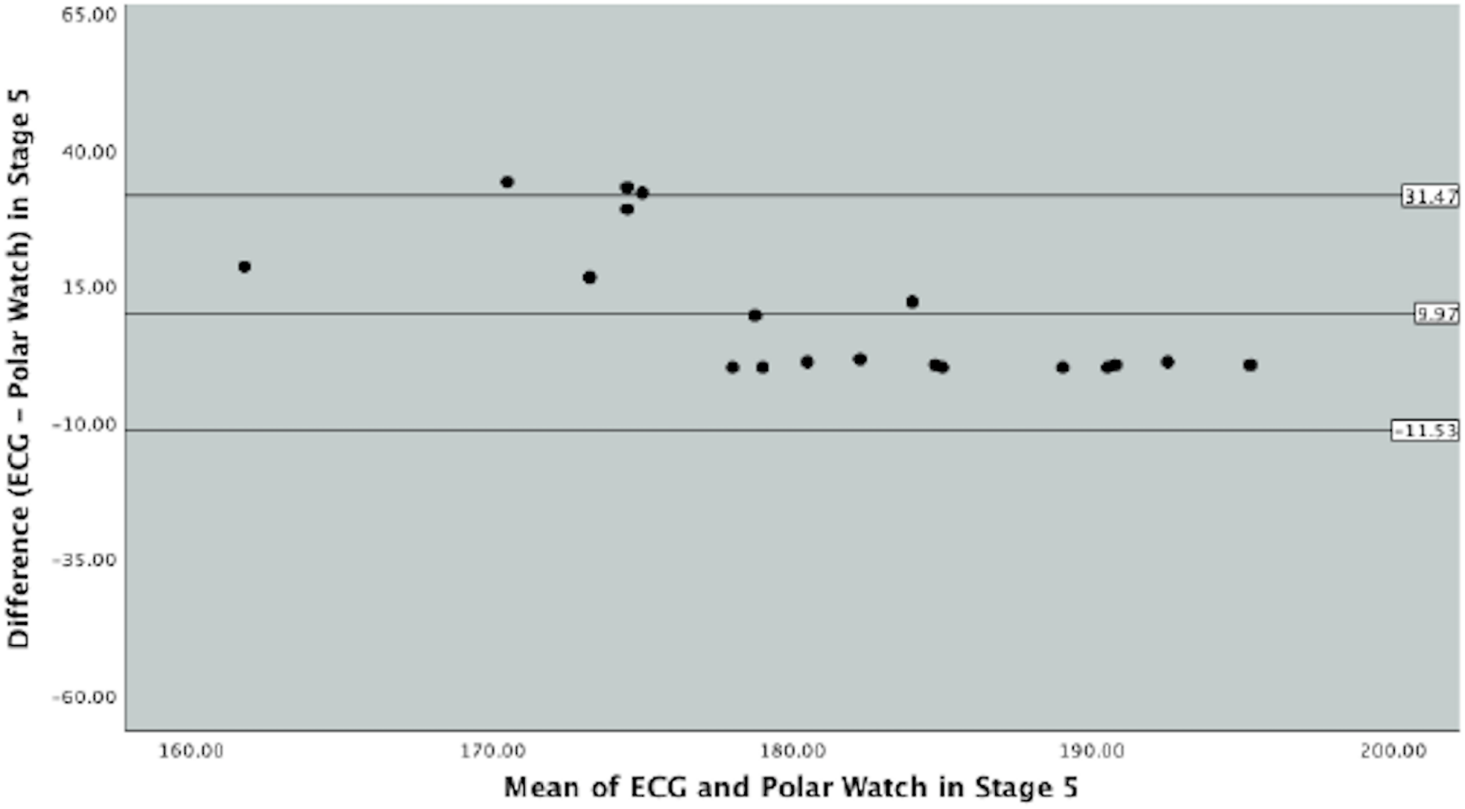
Bland–Altman Plot indicating mean difference and 90% limits of agreement between measurements from the Polar Vantage watch and ECG for HR measurement in Stage 3 of the Bruce Protocol.

Bland–Altman plots were produced to graphically depict the level of agreement between the ECG and PVM watch in measuring HR throughout the different exercise intensities in the protocol (Figs. 3, 4 and 5).

In Fig. 3, which represents Stage 1 of the Bruce Protocol, the mean difference between ECG and the PVM watch was 0.98 beats (SD 10.04, SE 1.89) and the upper and lower limits of agreements were 17.50 and −15.54 beats, respectively. Figure 3 displays majority of data points within the 90% limits of agreement with few outliers. Data points collected in Stage 1 varied between the ECG and PVM watch was 33.04 beats.

Stage 3 of the Bruce Protocol is represented by Fig. 4. The mean difference between the ECG and the PVM watch was 12.25 beats (SD 17.95, SE 3.39) with corresponding upper and lower limits of agreements of 41.78 and 17.28. This stage as seen in Fig. 4, graphically demonstrates a less cohesive set of data points as seen in the last stage with a larger variance in beats of 59.06.

Figure 5, graphically displays stage 5 of the Bruce protocol. The mean difference between the ECG and PVM watch was 9.97 (SD 13.07, SE 2.99) and the upper and lower limits of agreements were 31.47 and −11.53 respectively. Figure 4 details the majority of the data points within the 90% limits of agreement and close to the mean difference; however, data points can vary by 43.00 beats per min.

Intraclass correlation Coefficients were used to assess criterion validity between Polar and ECG HR data for each stage on Day 1 and Day 2. Good validity was seen for Day 1 and Day 2 for stage 0 (ICC = 0.83; 95% CI [0.63–0.92], ICC = 0.74; 95% CI [0.37–0.88]), stage 1 (ICC = 0.78; 95% CI [0.52–0.90], ICC = 0.88; 95% CI [0.74–0.95]), and stage 2 (ICC = 0.88; 95% CI [0.73–0.94], ICC = 0.80; 95% CI [0.40–0.92]). Poor validity was demonstrated on Day 1 and Day 2 for stages 3–5 (ICC < 0.50). Table 1 shows complete results for ICCs.

**Table 1:** Criterion Validity between ECG and the Polar Vantage watch throughout the Bruce Protocol.

Day 1		95% Confidence Interval		Day 2		95% Confidence Interval	
Stages	ICC	Lower Bound	Upper Bound	Stages	ICC	Lower Bound	Upper Bound
0	0.826	0.627	0.919	0	0.735	0.370	0.883
1	0.778	0.518	0.897	1	0.880	0.740	0.945
2	0.878	0.729	0.944	2	0.800	0.396	0.921
3	0.561	0.036	0.779	3	0.250	−0.273	0.603
4	0.623	0.183	0.826	4	0.141	0.426	0.538
5	0.313	−0.332	0.696	5	0.364	−0.315	0.728

## Discussion

### Main findings

The overall correlation between HR measurements from PVM watch vs ECG for both days combined was strong however results from ICC’s, and Bland–Altman Plots had wide limits of agreement (LoA), which raises concern about the safety of using the PVM watch in a clinical setting where precision is vital.

Our first main finding was that when combining data from Day 1 and 2, there is a strong correlation between HRs measured by the PVM watch and the ECG. This indicated that HR measures from the PVM watch and ECG were related; meaning that the watch can delineate between high and low HRs through a range of treadmill intensities. However, this high correlation does not necessarily mean these two measures agree. As explained by Bland & Altman (1986), correlation measures the strength of the relationship of variables and will be high if the points lie along any straight line. However, to agree, the points must lie along the line of equality (Bland & Altman, 1986). In the current literature, validity is concluded based upon results from correlation coefficients and mean biases of Bland–Altman Plots without including the implications of wide LoA in the conclusion (Etiwy et al., 2019; Jo et al., 2016; Delgado-Gonzalo et al., 2015). This can result in devices being deemed valid, when in fact, there are large differences in HR measurements for the individual (Cadmus-Bertram et al., 2017).

Looking closer at the variability of each stage, ICC values showed varied degrees of agreement between stages. Low to moderate intensities demonstrated moderate- good agreement, while high intensities showed poor to good agreement based upon upper and lower limits. This finding is consistent with other similar research. Boudreaux et al. (2018) determined the validity of eight HR monitors during a cycling regiment (up to 220 watts) and resistance exercise (10 RM) and found that HR measures from wearable devices were more accurate at rest and lower exercise intensities than at higher intensities. For example, Boudreaux et al. (2018) who investigated several wearable devices found ICCs ranging from 0.92 at rest to 0.12 at 200 watts on a cycle ergometer when using a FitBit. At rest, HR from most of the wearable devices had strong relationships to ECG values (*R* = 0.76–0.99). When exercise began as well as during each increase in exercise intensity, ICC were reduced in most devices (Boudreaux et al., 2018). Similar results were found by a study investigating the accuracy of the Fitbit Charge HR compared to Polar H6 HR monitor during free living condition by Gorny et al. (2017). Results demonstrated strong ICC coefficients for low intensity (ICC = 0.77(95% CI [0.55–0.87])) and moderate values for moderate to vigorous intensities (ICC = 0.56 (95% CI [0–0.79])) (Gorny et al., 2017).

Results from the Bland–Altman plots revealed a wide LoA seen across all intensities with increasing error as intensity increased. Although the mean differences were fairly small, there was large variation in HR throughout the protocol. Even at a low intensity, the error margin was large. In line with our finding, Wallen et al. (2016) reported limit of agreement (LoA) ranging between −27.30 bpm and 13.10 bpm in a study investigating the accuracy of four PPG-based watches at rest, walking/running and cycling. As intensity changed from low to moderate during stage 3, the largest mean difference and largest variation in bpm was observed. This is supported by a study investigating the validity of the Garmin Forerunner at different walking intensities. Bland–Altman plots from Claes et al. (2017) displayed a LoA range from −32.53 to 29.40 bpm, with largest error seen at moderate intensities.

The trend of observing decreasing agreement in HR as intensity increases may be explained by external factors to which PPG is sensitive. As the protocol progressed, the elevation and speed increased, resulting in higher levels of upper limb movement, higher transmitted vibration and transition to a flexed elbow arm swing pattern during running which occurred around stages 3–4 for most participants. This would support the hypothesis that Claes et al. (2017) made that running would result in less accurate HR measurements. Other studies have shown that PPG sensors are susceptible to poor accuracy during high intensity exercises that involve repetitive contractions of skeletal muscles which may result in decreased contact between the sensor and the skin (Rafolt & Gallasch, 2004; Allen, 2007; Spierer et al., 2015). Potential misalignment between the skin and optical sensor, variations in skin colour/skin tone, ambient light and poor tissue perfusion could also explain error (Alzahrani et al., 2015). Another factor which could influence PPG readings is perspiration (Maeda, Sekine & Tamura, 2011), however, care was taken to standardise room temperatures to 21 degrees Celsius as sweat is a factor that also affects PPG readings (Maeda, Sekine & Tamura, 2011).

### Strengths and Limitations

To the author’s knowledge, the PVM watch has not been validated against the gold standard of a medical grade ECG for determining rest and exercise HR which is a strength of this study. All testing was conducted in a highly controlled laboratory setting. The devices were assessed in young, healthy volunteers exercising in a standardised laboratory setting. The study methodology included the use of a standardised treadmill protocol (which included walking, jogging and running), tightly controlled room temperature and humidity as well as room lighting. The treadmill-stress testing system was stationary and not moved throughtout the testing period (or at any time), Additionally, our testing protocol was standardised such that we used four researchers performing the exact same role(s) during all testing sessions. The PVM watch was placed in a standard position, with the notches being recorded so the watch was worn on the same hand, same hand position, and same tightness for all testing. Although this study has a sufficient number of participants and included over 350 HR measurements over the 2 days of testing, it has its limitations. The study findings may vary for different categories of individuals such as cardiac patients, geriatric population or an unhealthy population with other co-morbidities. Although we accounted for participant factors including gender, age and body mass index, the narrow distribution of these characteristics does not enable us to rule out a potential factor on the accuracy of HR measurement. In addition, these results may not be indicative of the validity on other modes of exercise that are included on the watch (ex. biking, swimming, walking, resistance training, etc.). Another limitation was the time frame that participants returned to complete their second round of testing. Many participants noted symptoms of delayed onset of muscle soreness, especially in their gastrocnemius and soleus. This muscle fatigue or soreness may have factored into the data on the second day of testing influencing or skewing these results. Our participants were somewhat limited in that they were all Caucasian, however with varying skin tones only attributed to sun exposure. During our data analysis, we noticed a consistent outlier on the Bland–Altman plots which corresponded to an individual with a darker complexion. Upon removal of the outlier, values for the limits of agreement and mean difference changed. It is hypothesised that PPG signals may vary by skin tone and that failure to code for variation in skin complexity may have impacted the current study results (Fallow, Tarumi & Tanaka, 2013).

### Clinical applications

Results from our study can only be generalised to a generally healthy, young population due to the demographics of the participants. Given the large LoA and poor ICC’s at high intensities, caution is warranted from a clinical perspective if users are relying upon accurate HR readings for monitoring safe exercise intensities. There are no agreed upon standards for the level of error which is acceptable for monitoring clinical populations and depends upon the specific case due to the large spectrum of applications where HR monitoring can be used. One guideline from the American National Standard of Cardiac Monitors, Heart Rate Meters and Alarms has defined accuracy as ‘more readout error of no greater than ±10% of the input rate or ±5 bpm, whichever is greater’ (American National Standards Institute, 2002). Following these guidelines, our findings indicate the PVM watch is not appropriate for clinical use. Kottner et al. (2011) brings a discussion about acceptable limits of difference between the two measures of data points. The article deliberates that the decision is rather a clinical decision vs a statistical decision (Kottner et al., 2011). For populations with chronic heart conditions or need for exact HR measurement, these underlying pathologies could interfere with accuracy of HR measurement from the PVM watch. Cardiac patients with arrhythmias could be led astray from the HR readings and misinterpret their exertion levels which could be a detriment to their condition or pose a risk during physical activity or exercise. The levels of agreement between the ECG and PVM watch do not reach a safe enough clinical level of agreement to be used outside a healthy, recreational population.

### Where to from here

In order to determine safety for use in clinical populations studies involving such participants are needed. Larger sample sizes are needed to confirm our finding of large LoA at varying intensities. Additionally, further research should be conducted with a larger sample size of both genders, with genders stratified. Other modes of exercise need to be tested, such as cycle ergometer, rowing or resistance training, to investigate the affect of more and less upper limb movement to confirm the hypothesis that it is a factor that effects HR accuracy. Previous conclusions on accuracy of other devices cannot be generalised to all PPG devices. As more fitness trackers appear on the market, research needs to be conducted on accuracy of devices because PPG-based HR monitors may use different sensors, algorithms and infra-red wavelengths to detect HR (El-Amrawy & Nounou, 2015; Zhang, Pi & Liu, 2015). The impact of skin complexity on accuracy of PPG readings is poorly supported in the literature and more research to confirm variation in HR detection using this technology among different ethnicities is recommended (El-Amrawy & Nounou, 2015).

## Conclusions

Wrist worn fitness trackers have become increasingly popular throughout the fitness industry. With continual advancement in technology, it is important for researchers to continue to assess the validity of the trackers. This study revealed that the PVM watch has a strong correlation with the ECG throughout the entire Bruce Protocol, however, the LoA was widely dispersed as exercise intensities increase. Due to the large LoA between the ECG and PVM watch, the watch may not be advisable to use in settings where an accurate HR measure is needed. However, the watch could be utilised in a recreational population in which HR measures are not monitored as closely.

## Supplemental Information

10.7717/peerj.10893/supp-1 Supplemental Information 1 Dataset for comparing ECG and Polar Vantage Watch HR measurements during different exercise intensities.

## Additional Information and Declarations

### Data Availability

The following information was supplied regarding data availability: Raw data, including the participants’ ECG and Polar Vantage HR measurements during different stages of the Bruce Protocol Treadmill Test, are available as a Supplemental File.

## References

[ref-1] Abt G, Bray J, Benson A (2018). The validity and inter-device variability of the Apple WatchTM for measuring maximal heart rate. Journal of Sports Sciences.

[ref-2] Alharbi M, Straiton N, Smith S, Neubeck L, Gallagher R (2019). Data management and wearables in older adults: a systematic review. Maturitas.

[ref-3] Allen J (2007). Photoplethysmography and its application in clinical physiological measurement. Physiological Measurement.

[ref-4] Alzahrani A, Hu S, Azorin-Peris V, Barrett L, Esliger D, Hayes H, Akbare S, Achart J, Kuoch S (2015). A multi-channel optoelectronic sensor to accurately monitor heart rate against motion artefact during exercise. Sensors.

[ref-5] American National Standards Institute (2002). Cardiac monitors, heart rate meters, and alarms.

[ref-6] Bai Y, Hibbing P, Mantis C, Welk G (2018). Comparative evaluation of heart rate-based monitors: apple watch vs fitbit charge HR. Journal of Sports Sciences.

[ref-7] Bland JM, Altman DG (1986). Statistical methods for assessing agreement between two methods of clinical. Lancet.

[ref-8] Boudreaux B, Herbert E, Hollander D, Williams B, Cormier C, Naquin M, Wynnw G, Emilye G, Robertr K (2018). Validity of wearable activity monitors during cycling and resistance exercise. Medicine & Science in Sports & Exercise.

[ref-9] Bruce R, Kusumi F, Hosmer D (1973). Maximal oxygen intake and nomographic assessment of functional aerobic impairment in cardiovascular disease. American Heart Journal.

[ref-10] Cadmus-Bertram L, Gangnon R, Wirkus E, Thraen-Borowski K, Gorzelitz-Liebhauser J (2017). Accuracy of heart rate monitoring by some wrist-worn activity trackers. Annals of Internal Medicine.

[ref-11] Claes J, Buys R, Avila A, Finlay D, Kennedy A, Guldenring D, Budts W, Véronique C (2017). Validity of heart rate measurements by the Garmin Forerunner 225 at different walking intensities. Journal of Medical Engineering & Technology.

[ref-12] Collins T, Woolley SI, Oniani S, Pires IM, Garcia NM, Ledger SJ, Pandyan A (2019). Version reporting and assessment approaches for new and updated activity and heart rate monitors. Sensors.

[ref-13] Deborah Riebe JKE, Liguori G, Magal M, American College of Sports Medicine (2018). ACSM’s guidelines for exercise testing and prescription.

[ref-14] Delgado-Gonzalo R, Parak J, Tarniceriu A, Renevey P, Bertschi M, Korhonen I (2015). Evaluation of accuracy and reliability of PulseOn optical heart rate monitoring device. 26th Annual International Conference of the IEEE Engineering in Medicine and Biology Society.

[ref-15] Dooley E, Golaszewski N, Bartholomew J (2017). Estimating accuracy at exercise intensities: a comparative study of self-monitoring heart rate and physical activity wearable devices. JMIR mHealth and uHealth.

[ref-16] El-Amrawy F, Nounou MI (2015). Are currently available wearable devices for activity tracking and heart rate monitoring accurate, precise, and medically beneficial?. Healthcare Informatics Research.

[ref-17] Essa.org.au (2019). Adult pre-screening tool. https://www.essa.org.au/Public/ABOUT_ESSA/Adult_Pre-Screening_Tool.aspx.

[ref-18] Etiwy M, Akhrass Z, Gillinov L, Alashi A, Wang R, Blackburn G, Gillinov SM, Phelan D, Gillinov AM, Houghtaling PL, Javadikasgari H, Desai MY (2019). Accuracy of wearable heart rate monitors in cardiac rehabilitation. Cardiovascular Diagnosis and Therapy.

[ref-19] Fallow BA, Tarumi T, Tanaka H (2013). Influence of skin type and wavelength on light wave reflectance. Journal of Clinical Monitoring and Computing.

[ref-20] Fleiss JL, Levin B, Paik MC (2003). Statistical methods for rates and proportions.

[ref-21] Gillinov S, Etiwy M, Wang R, Blackburn G, Phelan D, Gillinov A, Houghtaling P, Javadikasgari H, Desai M (2017). Variable accuracy of wearable heart rate monitors during aerobic exercise. Medicine & Science in Sports & Exercise..

[ref-22] Gorny A, Liew S, Tan C, Müller-Riemenschneider F (2017). Fitbit charge HR wireless heart rate monitor: validation study conducted under free-living conditions. JMIR mHealth and uHealth.

[ref-23] Haghayegh S, Khoshnevis S, Smolensky M, Diller K (2019). Accuracy of PurePulse photoplethysmography technology of Fitbit Charge 2 for assessment of heart rate during sleep. Chronobiology International.

[ref-24] Hernando D, Roca S, Sancho J, Alesanco Á, Bailón R (2018). Validation of the apple watch for heart rate variability measurements during relax and mental stress in healthy subjects. Sensors.

[ref-25] Jo E, Lewis K, Directo D, Kim M, Dolezal B (2016). Validation of biofeedback wearables for photoplethysmographic heart rate tracking. Journal of Sports Science and Medicine.

[ref-26] Khushhal A, Nichols S, Evans W, Gleadall-Siddall D, Page R, O’Doherty A, Carroll S, Ingle L, Abt G (2017). Validity and reliability of the apple watch for measuring heart rate during exercise. Sports Medicine International Open.

[ref-27] Koo TK, Li MY (2016). A guideline of selecting and reporting intraclass correlation coefficients for reliability research. Journal of Chiropractic Medicine.

[ref-28] Koo TK, Mae LY (2017). A guideline of selecting and reporting intraclass correlation coefficients for reliability research. Journal of Chiropractic Medicine.

[ref-29] Kottner J, Audige L, Brorson S, Donner A, Gajewski BJ, Hróbjartsson A, Roberts C, Shoukri M, Streiner DL (2011). Guidelines for reporting reliability and agreement studies (GRRAS) were proposed. International Journal of Nursing Studies.

[ref-30] Lee C, Gorelick M (2011). Validity of the smarthealth watch to measure heart rate during rest and exercise. Measurement in Physical Education and Exercise Science.

[ref-31] Leth S, Hansen J, Nielsen O, Dinesen B (2017). Evaluation of commercial self-monitoring devices for clinical purposes: results from the future patient trial, phase I. Sensors.

[ref-32] Lexell JE, Downham DY (2005). How to assess the reliability of measurements in rehabilitation. American Journal of Physical Medicine & Rehabilitation.

[ref-33] Maeda Y, Sekine M, Tamura T (2011). The advantages of wearable green reflected photoplethysmography. Journal of Medical Systems.

[ref-34] Maeda Y, Sekine M, Tamura T (2011). Relationship between measurement site and motion artifacts in wearable reflected photoplethysmography. Journal of Medical Systems.

[ref-35] Mukaka MM (2012). A guide to appropriate use of Correlation coefficient in medical research. Malawi Medical Journal.

[ref-36] Nelson B, Allen N (2019). Accuracy of consumer wearable heart rate measurement during an ecologically valid 24-hour period: intraindividual validation study. JMIR Mhealth Uhealth.

[ref-37] Phaneuf A (2019). Latest trends in medical monitoring devices and wearable health technology. Business Insider.

[ref-38] Polar Australia (2020). How to track heart rate?: smart coaching. https://www.polar.com/au-en/smart-coaching/polar-heart-rate-measurement-technology.

[ref-39] Polar USA (2020). Polar vantage M: GPS running & multisport watch with wrist-based heart rate. Mobile Industry Reviews.

[ref-40] Pope ZC, Lee JE, Zeng N, Gao Z (2019). Validation of four smartwatches in energy expenditure and heart rate assessment during exergaming. Games for Health Journal.

[ref-41] Pothitos A (2016). The history of the: smartwatch. Mobile industry review. http://www.mobileindustryreview.com/tag/old-smartwatches.

[ref-42] Powierza CS, Clark MD, Hughes JM, Carbeiro KA, Mihalik JP (2017). Validation of a self-monitoring tool for use in exercise therapy. American Academy of Physical Medicine and Rehabilitation.

[ref-43] Queensland Health (2019). Exercise stress testing cardiac sciences. https://www.health.qld.gov.au/__data/assets/pdf_file/0024/147624/qh-gdl-392.pdf.

[ref-44] Rafolt D, Gallasch E (2004). Influence of contact forces on wrist photoplethysmography—prestudy for a wearable patient monitor. Biomedizinische Technik/Biomedical Engineering.

[ref-45] Reddy RK, Pooni R, Zaharieva DP, Senf B, El Youssef J, Dassau E, Doyle FJ, Clements MA, Rickels MR, Patton SR, Castle JR, Riddell MC, Jacobs PG (2018). Accuracy of wrist-worn activity monitors during common daily physical activities and types of structured exercise: evaluation study. JMIR mHealth and uHealth.

[ref-46] Shcherbina A, Mattsson CM, Waggott D, Salisbury H, Christle JW, Hastie T, Wheeler MT, Ashley EA (2017). Accuracy in wrist-worn, sensor-based measurements of heart rate and energy expenditure in a diverse cohort. Journal of Personalized Medicine.

[ref-47] Shin C, Shin H, Lee M (2011). Relations between ac-dc components and optical path length in photoplethysmography. Journal of Biomedical Optics.

[ref-48] Spierer DK, Rosen Z, Litman LL, Fujii K (2015). Validation of photoplethysmography as a method to detect heart rate during rest and exercise. Journal of Medical Engineering & Technology.

[ref-49] Stahl S, An H, Dinkel DM, Noble JM, Lee J (2016). How accurate are the wrist-based heart rate monitors during walking and running activities? Are they accurate enough?. BMJ Open Sport & Exercise Medicine.

[ref-50] Statista (2020). Wearables–Australia: Statista Market Forecast. https://www.statista.com/outlook/319/107/wearables/australia#market-arpu.

[ref-51] Stove MP, Haucke E, Nymann ML, Sigurdsson T, Larsen BT (2019). Accuracy of the wearable activity tracker Garmin Forerunner 235 for the assessment of heart rate during rest and activity. Journal of Sports Sciences.

[ref-52] Tedesco S, Sica M, Ancillao A, Timmons S, Barton J, O’Flynn B (2019). Accuracy of consumer-level and research-grade activity trackers in ambulatory settings in older adults. PLOS ONE.

[ref-53] Thiebaud RS, Funk MD, Patton JC, Massey BL, Shay TE, Schmidt MG, Giovannitti N (2018). Validity of wrist-worn consumer products to measure heart rate and energy expenditure. Digit Health.

[ref-54] Thompson W (2015). Worldwide survey of fitness trends for 2016. ACSMs Health & Fitness Journal.

[ref-55] Thompson W (2019). Worldwide survey of fitness trends for 2020. ACSMʼs Health & Fitness Journal.

[ref-56] Thomson EA, Nuss K, Comstock A, Reinwald S, Blake S, Pimentel RE, Tracy BL, Li K (2019). Heart rate measures from the Apple Watch, Fitbit Charge HR 2, and electrocardiogram across different exercise intensities. Journal of Sports Sciences.

[ref-57] Wallen MP, Gomersall SR, Keating SE, Wisløff U, Coombes JS, Calbet JAL (2016). Accuracy of heart rate watches: implications for weight management. PLOS ONE.

[ref-58] Wang Z, Fu S (2016). Evaluation of a strapless heart rate monitor during simulated flight tasks. Journal of Occupational & Environmental Hygiene.

[ref-59] Welch Allyn (2018). XScribe™ cardiac stress testing system. https://www.welchallyn.com/en/products/categories/cardiopulmonary/stress-test-systems/xscribe.html.

[ref-60] Zhang Z, Pi Z, Liu B (2015). TROIKA: a general framework for heart rate monitoring using wrist-type photoplethysmographic signals during intensive physical exercise. IEEE Transactions on Biomedical Engineering.

